# Improved High-Quality Draft Genome Sequence and Annotation of *Burkholderia contaminans* LMG 23361^T^

**DOI:** 10.1128/genomeA.00245-17

**Published:** 2017-04-20

**Authors:** Ji Young Jung, Youngbeom Ahn, Ohgew Kweon, John J. LiPuma, David Hussong, Bernard S. Marasa, Carl E. Cerniglia

**Affiliations:** a Division of Microbiology, National Center for Toxicological Research, U.S. Food and Drug Administration, Jefferson, Arkansas, USA; b Department of Pediatrics and Communicable Diseases, University of Michigan, Ann Arbor, Michigan, USA; c ValSource, LLC, Downingtown, Pennsylvania, USA; d Division of Microbiology Assessment, Office of Pharmaceutical Quality, Center for Drug Evaluation and Research, U.S. Food and Drug Administration, Silver Spring, Maryland, USA

## Abstract

*Burkholderia contaminans* LMG 23361 is the type strain of the species isolated from the milk of a dairy sheep with mastitis. Some pharmaceutical products contain disinfectants such as benzalkonium chloride (BZK) and previously we reported that *B. contaminans* LMG 23361^T^ possesses the ability to inactivate BZK with high biodegradation rates. Here, we report an improved high-quality draft genome sequence of this strain.

The *Burkholderia cepacia* complex (BCC) is a bacterial group that is ubiquitous in the environment and capable of infecting immunocompromised individuals as opportunistic human pathogens, especially in persons with cystic fibrosis (CF) (1–3). *Burkholderia contaminans* is an emerging BCC species in CF infections and is increasingly being isolated from CF patients (4–7). *B. contaminans* also has been found as a contaminant of pharmaceutical and personal care products (PPCPs) and linked to outbreaks (8–11). Recently, we reported that *B. contaminans* HI3429 (=LMG 23361^T^) is able to degrade benzalkonium chloride (BZK), one of major antiseptic formulations for PPCPs, at higher levels than the other BCC strains (12).

*B. contaminans* LMG 23361^T^ was isolated from the milk of dairy sheep with mastitis in Spain (4, 13, 14). This genome was initially sequenced in 2015 and assembled into 18 contigs (13). To close the significant genomic gaps and to increase qualitative and quantitative genomic resolution focused on antiseptic resistance, we resequenced the *B. contaminans* LMG 23361^T^ genome.

The whole genome was sequenced at Macrogen Corporation (Rockville, MD, USA) with a combination of the Illumina HiSeq platform with 2 × 100-bp reads and the Pacific Biosciences (PacBio) RSII SMRT sequencing platform using a 20-kb SMRTbell template library. Approximately 3,031.4 Gb (331.4570-fold coverage) with 30,014,102 paired-end reads and approximately 547 Mb (59.8609-fold coverage) with 99,320 reads were generated from the Illumina HiSeq and PacBio sequencing, respectively. The resulting reads were assembled using HGAP3 in PacBio’s SMRT portal and Pilon version 1.17. SeqMan (DNA Star, USA) was used for reassembling the contigs. The assembled sequence was submitted to the NCBI Prokaryotic Genome Annotation Pipeline (PGAP) for annotation. The locus tag prefix was set as “BED46.”

The genome sequencing and assembly strategy led to a draft genome of four contigs containing 10,352,616 bp with a 65.7% G+C content. NCBI PGAP predicted 9,054 protein-coding genes, 25 rRNAs (seven 5S, nine 16S , and nine 23S), 74 tRNAs, four noncoding RNAs, and 115 pseudogenes. Our previous study showed that the BCC strains tested, including *B. contaminans* LMG 23361^T^ and *B. cenocepacia* AU1054, share intrinsic and widespread BZK resistance mechanisms, including efflux pumps and enzymatic inactivation via biodegradation (12). Comparative genomic analysis of strain LMG 23361^T^ and strain AU1054 by local BLAST revealed 51 conserved orthologous genes from strain LMG 23361^T^; 27 orthologous genes encoding degradation enzymes involved in the C-N cleavage (N-dealkylation) and the subsequent degradation of the corresponding benzyldimethylamine (BDMA) via benzoate and alkyl aldehyde via fatty acid, as well as 24 genes encoding proteins functioning for efflux pumping and membrane permeability, are completely conserved in the genome (12). In conclusion, our systematic endeavor of genome sequencing, reassembling, and functional annotation of *B. contaminans* LMG 23361^T^ enabled us to increase the quantity and quality of genomic information, significantly reduce the number of contigs from 18 to four with high coverage, and provide a practical annotation of the genes responsible for antiseptic resistance.

## Accession number(s).

This draft genome sequence has been deposited at DDBJ/ENA/GenBank under the accession number MCAU00000000. The version described in this paper is the second version, MCAU02000000.
